# Design and Application of Biosafe Coronavirus Engineering Systems without Virulence

**DOI:** 10.3390/v16050659

**Published:** 2024-04-24

**Authors:** Guoqiang Wu, Qiaoyu Li, Junbiao Dai, Guobin Mao, Yingxin Ma

**Affiliations:** 1 CAS Key Laboratory of Quantitative Engineering Biology, Guangdong Provincial Key Laboratory of Synthetic Genomics and Shenzhen Key Laboratory of Synthetic Genomics, Shenzhen Institute of Synthetic Biology, Shenzhen Institute of Advanced Technology, Chinese Academy of Sciences, Shenzhen 518055, China; gq.wu@siat.ac.cn (G.W.); lqy_10031@163.com (Q.L.); junbiao.dai@siat.ac.cn (J.D.); 2 School of Pharmacy, Faculty of Medicine, Macau University of Science and Technology, Macau SAR 999078, China; 3 College of Biotechnology, Tianjin University of Science and Technology, Tianjin 300457, China; 4 Shenzhen Branch, Guangdong Laboratory for Lingnan Modern Agriculture, Key Laboratory of Synthetic Biology, Ministry of Agriculture and Rural Affairs, Agricultural Genomics Institute at Shenzhen, Chinese Academy of Agricultural Sciences, Shenzhen 518120, China

**Keywords:** coronavirus, biosafety, pseudovirus, trans-complementation system, application

## Abstract

In the last twenty years, three deadly zoonotic coronaviruses (CoVs)—namely, severe acute respiratory syndrome coronavirus (SARS-CoV), Middle East respiratory syndrome coronavirus (MERS-CoV), and SARS-CoV-2—have emerged. They are considered highly pathogenic for humans, particularly SARS-CoV-2, which caused the 2019 CoV disease pandemic (COVID-19), endangering the lives and health of people globally and causing unpredictable economic losses. Experiments on wild-type viruses require biosafety level 3 or 4 laboratories (BSL-3 or BSL-4), which significantly hinders basic virological research. Therefore, the development of various biosafe CoV systems without virulence is urgently needed to meet the requirements of different research fields, such as antiviral and vaccine evaluation. This review aimed to comprehensively summarize the biosafety of CoV engineering systems. These systems combine virological foundations with synthetic genomics techniques, enabling the development of efficient tools for attenuated or non-virulent vaccines, the screening of antiviral drugs, and the investigation of the pathogenic mechanisms of novel microorganisms.

## 1. Introduction

Coronaviruses (CoVs) have many hosts, and some CoVs can spread across species. Therefore, they can infect humans from different animals and then achieve human-to-human transmission. Every outbreak of a novel human coronavirus triggers panic and social disorder due to its potent infectivity and high fatality rates. To date, seven types of CoV have been found to infect humans and cause respiratory diseases: three highly pathogenic CoVs, namely severe acute respiratory syndrome CoV (SARS-CoV), SARS-CoV-2, and Middle East respiratory syndrome CoV (MERS-CoV), and four low pathogenic CoVs, namely human CoV 229E (HCoV-229E), human CoV OC43 (HCoV-OC43), human CoV NL63 (HCoV-NL63), and human CoV HKU1 (HCoV-HKU1) [1].

### 1.1. Coronavirus Outbreaks

In 1937, Beaudette and Hudson et al. isolated a highly pathogenic virus from the embryos of chickens whose respiratory systems had been infected [2]. This highly infectious virus was the first CoV to be isolated from an organism and was named Infectious Bronchitis Virus (IBV) [2]. Then, in 1965, the first CoV to infect humans was isolated from the upper respiratory tract of common cold patients and named the B814 strain [3]. This strain was cultured using human embryonic trachea culture. Further research indicated that B814 was significantly correlated with the 229E strain isolated and identified in 1967, officially renamed as HCoV-229E [4]. In 1967, another human-infecting CoV strain, HCoV-OC43, was isolated [5]. HCoV-229E and HCoV-OC43 cause relatively mild common colds; however, the symptoms can become severe in infants, the elderly, and immunocompromised individuals [6,7].

**Figure 1:**
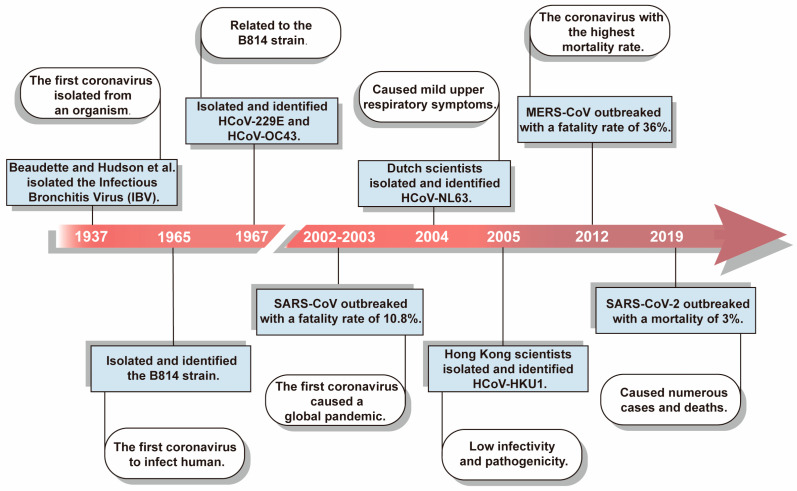
Timeline of key events related to coronavirus outbreaks. So far, seven coronaviruses that infect humans have been identified. Among them, HCoV-229E, HCoV-OC43, HCoV-NL63, and HCoV-HKU1 only cause mild respiratory diseases. Conversely, the pathogenicity and infectivity of SARS-CoV, MERS-CoV, and SARS-CoV-2 are so high that their transmission was widespread, causing deaths worldwide.

Before 2003, CoVs were believed to cause only mild symptoms. However, the SARS-CoV outbreak from 2002 to 2003 altered this view [8]. SARS-CoV, transmitted via close air droplets and contact, affected 32 countries, caused 8422 infections and 916 deaths, and had a fatality rate of 10.8% [9]. Furthermore, in 2004, Dutch scientists isolated and identified HCoV-NL63 from an infant with bronchitis and conjunctivitis [10]. The following year, scientists in Hong Kong isolated and identified HCoV-HKU1 in the nasopharyngeal secretions of patients with acute pneumonia [11]. It has been indicated that HCoV-NL63 and HCoV-HKU1 can cause mild to severe lower respiratory tract infections in infants, children, and adults, thereby inducing tracheitis, bronchitis, pneumonia, and other diseases [12]. In 2012, Saudi Arabia reported the emergence of MERS-CoV, a new CoV that shared multiple characteristics with SARS-CoV, caused lower respiratory tract disease, spread to multiple regions, and had a fatality rate of up to 36% [13,14,15,16,17,18]. In 2019, SARS-CoV-2 was discovered as the seventh CoV capable of infecting humans and was proven to be more infectious than SARS-CoV [19,20,21]. SARS-CoV-2 caused the global COVID-19 pandemic, triggering public panic, economic losses, and numerous deaths [22,23] (Figure 1). Currently, the vaccine’s effectiveness against viral mutations and the duration of antibody protection are uncertain; therefore, the urgent need for effective therapeutic drugs is evident [24,25,26].

The World Health Organization (WHO) updated and released laboratory biosafety guidelines related to SARS-CoV-2 (COVID-19) on 11 March 2024. Non-propagative diagnostic lab work, such as sequencing and nucleic acid amplification tests, requires a risk assessment and biosafety level 2 (BSL-2) protocols. Propagative tasks, such as virus culture and animal studies, need local risk assessment and BSL-2 (or higher) precautions. Handling high concentrations of live SARS-CoV-2 variants or large volumes of infectious material mandates trained personnel in BSL-3 compliant labs following rigorous risk assessments. Although SARS-CoV-2 has evolved to pose a lesser threat to humans due to mutations, permitting its handling in BSL-2 labs, other CoVs, such as SARS-CoV and MERS-CoV, still require BSL-3 facilities. This distinction will not alter the overall risk assessment of the coronavirus family.

### 1.2. The Morphology and Genomic Structure of Coronaviruses

**Figure 2:**
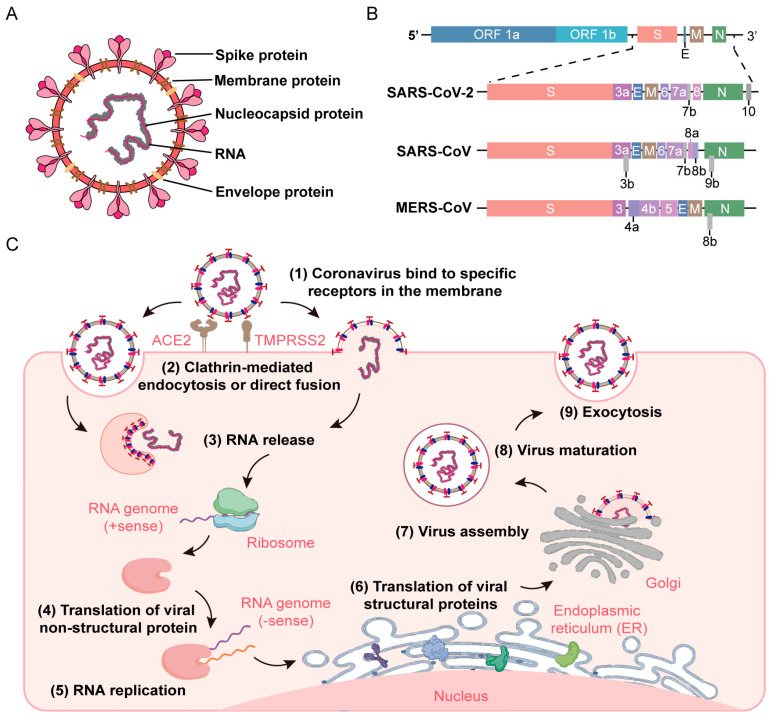
Coronavirus virion genomic structure and life cycle. (**A**) The structure of coronavirus particles, containing four structural proteins, namely Spike (S), Membrane (M), Nucleocapsid (N), and Envelope (E) protein, all of which are encoded within the 3′ end of the viral genome. The non-segmented positive (+)-sense single stranded RNA is surrounded by the proteins. (**B**) The genome structure of SARS-CoV-2, SARS-CoV, and MERS-CoV. The dotted line displays the critical difference among CoVs. (**C**) An overview for the life cycle of coronavirus in the host cell. When (1) recognized by cell receptors (ACE2/TMPRSS2), virus particles entered host cells by (2) direct fusion of the viral and cell host surface or through endocytosis. And (3) viral (+) ssRNA was released into the cytoplasm. (4) Non-structural proteins were translated by host ribosome with the template of genome RNA, and (5) (+) ssRNA replicated, generating the (−) ssRNA. The structural proteins were (6) translated and (7) assembled in the endoplasmic reticulum and Golgi. Finally, the virus particles were (8) matured and (9) released via exocytosis.

The genus *Coronavirus* belongs to the order Nidovirales and the family Coronaviridae. The CoVs are the largest known RNA viruses, with membranes comprising spikes arranged in a regular crown pattern, hence the name CoV [12,27,28] (Figure 2A). Mature CoVs are mostly round with a diameter of 70 to 120 nm; however, some can be oval or mildly polymorphic [1,29]. They are highly diverse enveloped viruses with a single-stranded, positive-sense RNA genome of 26–32 kilobases (kbs) [30]. Their genome has a methylated cap structure at the 5’ end and a polyA tail at the 3’ end, which is very similar to the structure of messenger RNA (mRNA) and an important basis for the use of viral genomic RNA as a translation template [31]. Furthermore, CoVs have similar genome organization and gene expression, with open reading frame 1a/b (ORF1a/b) encoding 16 non-structural proteins (NSPs; NSP1–NSP16) at the 5’ end. Other ORFs at the 3’ end encode structural proteins, including spike (S), membrane (M), envelope (E), and nucleocapsid (N) proteins [32] (Figure 2B).

### 1.3. The Life Cycle of Coronaviruses

CoVs specifically interact with the host’s cell receptors, such as angiotensin-converting enzyme 2 (ACE2), and factors such as cell surface serine protease (TMPRSS2), promoting its fusion and endocytosis to enter the cells [33]. After entry, viral genomic RNA is uncoated and released, prompting the translation of ORF1a/b to obtain polyproteins (pp1a and pp1ab), which are further processed to produce the various NSPs necessary for viral replication. The translated structural proteins are translocated to the endoplasmic reticulum membrane and Golgi apparatus, interact with N proteins encapsulating the genomic RNA, and then bud into the secretory vesicular. Lastly, virions are secreted from infected cells via exocytosis [34] (Figure 2C).

### 1.4. The Necessity of Developing Biosafety Coronavirus Engineering Systems

In the last two decades, three highly pathogenic human CoVs (SARS-CoV, MERS-CoV, and SARS-CoV-2) have posed a significant threat to global health security. They not only have high incidence and mortality rates but also lack effective targeted drugs and therefore warrant further study [35]. To protect researchers exposed to such pathogenic organisms, the biosafety assessment of these novel viruses is essential, including the risk levels of pathogens and biosafety laboratories operating on them. *The Laboratory Biosafety Manual (4th Edition)* issued by the World Health Organization (WHO) lists highly infectious microorganisms with high individual and low/high community risks that need to be studied in biosafety level 3/4 labs (BSL-3/4) [36]. Furthermore, according to the 6th edition of *Biosafety in Microbiological and Biomedical Laboratories* (BMBL) by the Centers for Disease Control and Prevention (CDC), SARS-CoV, MERS-CoV, and SARS-CoV-2 are highly pathogenic viruses in the *Coronaviridae* family. Therefore, the identification and investigation of relevant pathogens, clinical samples, and cell culture–produced viruses should be carried out in BSL-3 conditions [37]. The establishment of a biosafe laboratory has greatly improved the safety of research on pathogenic microorganisms and the response to emergencies that threaten biosafety. However, the use of high–biosafety level labs has many limitations, such as high operating and maintenance costs, limited experimental space, and uneven distribution of labs [38].

With the continuous spread of COVID-19 and the possible risk of unknown outbreaks, the scarcity of resources in high–biosafety level labs significantly limit essential research, which has resulted in delayed vaccine development and antiviral drug screening [39]. Therefore, biosafe CoV engineering systems without virulence have been developed, allowing the use of conventional labs for partial research on these highly infectious viruses. Compared with wild-type viruses, engineered virus particles have better biological safety and can be worked on in BSL-2 labs [40,41,42]. Moreover, these engineered viruses are being widely used in receptor recognition, virus inhibition, antibody evaluation, and vaccine development, providing an important platform for the development of virology. In addition, the quantitative analysis of engineered virus particles is easier due to the introduction of different reporter genes [43].

## 2. Whole Genome Synthesis of Coronavirus

The rapid development of the field of DNA sequencing and synthesis has substantially helped to further understand the characteristics of organisms. Furthermore, with the use of genetic data, various viral diseases can be effectively prevented and treated [44]. The de novo synthesis of viral genomes without natural templates is essential for virological research, providing new technical tools for comprehensively understanding the function and expression of viral genes, as well as their pathogenic mechanisms [45]. Moreover, with this technology, large-scale genetic modifications of the viral genome can be performed, which is not achievable by traditional molecular biology methods. The CoV, as the largest single-stranded RNA virus, has always been a hotspot in virus genome synthesis, mainly relying on synthetic genomics and reverse genetics [46,47]. However, the whole genome assembly of CoVs is extremely challenging because of their genome size, the toxicity of their genomic region, and the presence of mutations and deletions in the genome sequence.

### 2.1. Synthetic Genomics

Synthetic genomics mainly includes the design, synthesis, assembly, and transplantation of genomes. It allows large-scale modification and synthesis of an organism’s genome [48]. Genome synthesis using genome sequences can reconstruct viruses or other organisms without natural templates [49]. The first complete virus genome synthesized was poliovirus. Eckard Wimmer et al. rescued poliovirus type 1 (Mahoney) [PV1(M)] through chemical synthesis, marking the beginning of a new era in biology [50]. Furthermore, in 2008, Denison et al. successfully synthesized a 29.7 kb cDNA of bat SARS-like CoV (Bat-SCoV) and transformed it into an infectious clone. This clone could infect cells and mice by exchanging its receptor binding domain (RBD) with SARS-CoV-RBD [51]. De novo genome synthesis allows on-demand genome synthesis and modification, which is different from wild-type genomes.

### 2.2. Reverse Genetics

Reverse genetics is a method to study a genome’s structure and function by modifying specific genes or non-coding nucleic acids based on biological genetic information, such as by targeting gene mutations or gene insertions/deletions/replacements [47]. Furthermore, it is not only used for the comprehensive analysis of molecular characteristics, pathogenesis, and virus–host interactions but can also help develop novel vaccines by directly manipulating RNA virus genomes. Moreover, it has been widely employed for the whole genome synthesis of viruses, including SARS-CoV, MERS-CoV, and SARS-CoV-2, making it an essential tool in virological research. The basic construction strategy of reverse genetics is to (i) obtain all DNA fragments of the virus genome via RT-PCR or chemical synthesis; (ii) assemble these fragments into full-length complementary DNA (cDNA) by targeted RNA recombination, in vitro ligation, transformation-associated recombination (TAR), or circular polymerase extension reaction (CPER); and then (iii) transfect the acquired cDNA into susceptible cells to initiate the viral life cycle and rescue the infectious virus [52,53,54,55].

#### 2.2.1. Targeted RNA Recombination

**Figure 3:**
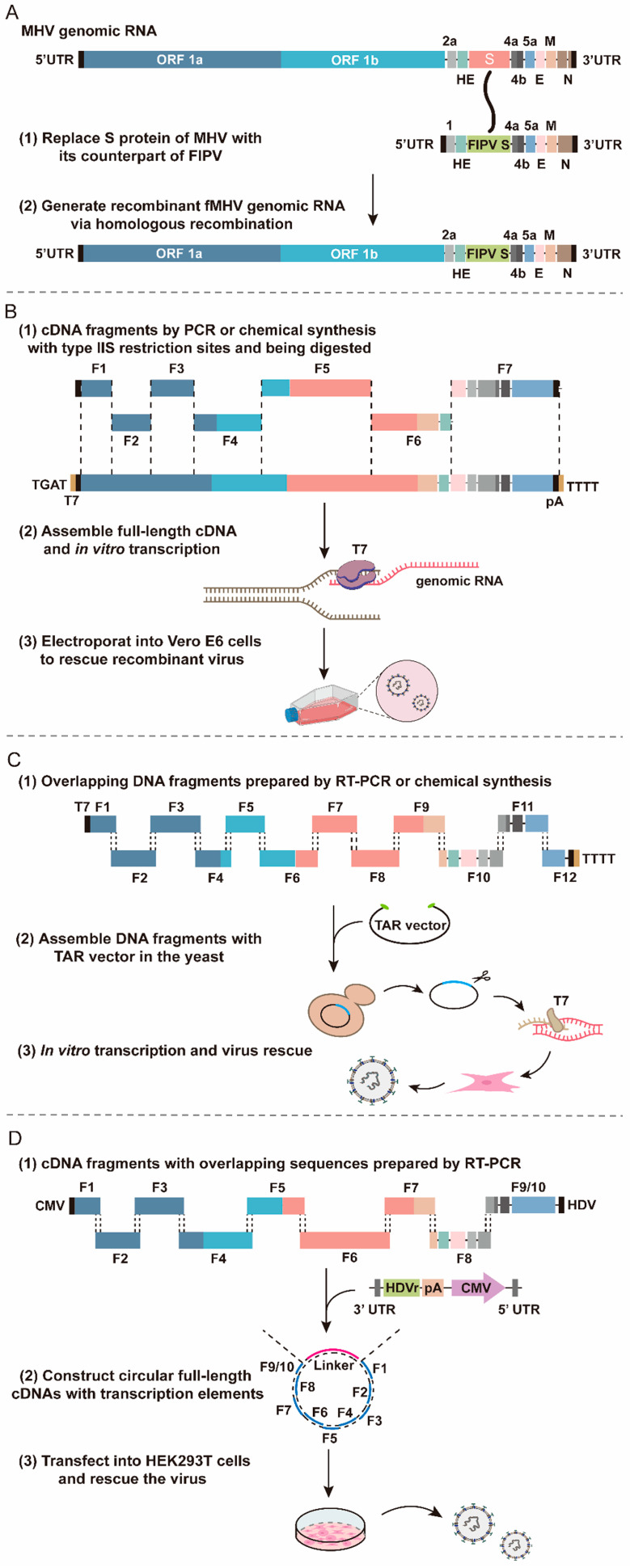
The common strategies of reverse genetics. (**A**) Targeted RNA recombination. The synthetic donor RNA containing the S gene of feline infectious peritonitis virus (FIPV) was transfected into mouse L2 cells, which were infected with the thermolabile MHV N gene deletion mutant. A crossover event occurs within the HE gene fragment during targeted recombination. Recombinant MHV genomic RNA containing the FIPV S gene is produced. (**B**) In vitro ligation. The specific sites in every fragment were recognized and cleaved by IIS restriction endonucleases, which were assembled into full-length cDNA. These were then transcribed in vitro and SARS-CoV-2 RNA was transfected in cells to rescue the virus. (**C**) TAR. Overlapping DNA fragments were transmitted into yeast with linearized TAR vector, and all DNA fragments were assembled by homologous recombination to generate the YAC vector containing the viral full-length cDNA. (**D**) CPER. Circular viral cDNA with transcription elements was constructed, which include CMV promoter, HDV ribozyme (HDVr), and transcriptional terminator sequence (polyA). The SARS-CoV-2 virus was rescued after being transfected in the package cells.

Targeted RNA recombination utilizes the high recombination efficiency of the CoV genome with homologous RNA sequences. This technique introduces specific alteration by recombination of the donor-synthesized RNA with the recipient CoV genome to enable reverse selection of the CoV [56,57,58]. The artificially synthesized RNA covers the 3’ end of the CoV genome, with a size of approximately 10 kb (Figure 3A). This synthesized RNA is then transfected into host cells infected with the CoV, and the recombinant virus is purified by controlling the culture temperature [57,59].

#### 2.2.2. In Vitro Ligation

In vitro ligation is a common reverse genetics technique that uses type IIS restriction endonucleases to sequentially assemble DNA fragments into full-length cDNA. For generating stable and infectious viral genomic RNA, it is essential to linearize cDNA with T7 promoter at the 5′ end and polyA tail at the 3′ end [60,61]. T7 RNA polymerase is used for in vitro RNA transcription and transfection into cells to rescue the positive-sense RNA virus [62] (Figure 3B). This method was first applied to the assembly of the transmissible gastroenteritis virus (TGEV) genome, where small DNA fragments were flanked by natural or engineered specific restriction sites for the precise assembly of full-length cDNA [46]. Moreover, in vitro ligation has also been employed to construct the genomes of other CoVs, such as IBV, SARS-CoV, Bat-SCoV, HCoV-NL63, and MERS-CoV [46,63,64,65,66]. Baric et al. and Shi et al. both used an in vitro ligation strategy to acquire SARS-CoV-2 infectious clones with replication capabilities similar to clinical isolates [67,68].

#### 2.2.3. Transformation-Associated Recombination

The TAR is a single-step assembly method that uses a highly efficient homologous recombination system of yeast to assemble multiple DNA fragments with overlapping sequences [69]. It not only assembles shorter DNA fragments into longer DNA fragments but can also assemble the entire genome [70]. For assembly, the TAR vector and multiple viral subgenomic fragments are transformed into yeast, and then the linearized TAR vector is homologously recombined with the yeast genome. The virus rescue is detected by yeast artificial chromosome (YAC) carrying viral cDNA (Figure 3C). TAR cloning technology has been successfully applied to construct infectious clones of Bat-SCoV, SARS-CoV, and SARS-CoV-2 [51,71,72].

#### 2.2.4. Circular Polymerase Extension Reaction

Circular polymerase extension reaction (CPER) is a gene assembly technique based on polymerase chain reaction (PCR), which can quickly generate viral infectious cDNA (Figure 3D). CPER does not require in vitro plasmid construction and RNA transcription [73]. Amarilla et al. and Torii et al. performed CPER to rescue infectious clones of SARS-CoV-2 [74,75]. They used specific primers to amplify viral DNA fragments and linker fragments encoding viral 5′ and 3′ terminus, hepatitis delta virus ribozyme, bovine growth hormone, cytomegalovirus (CMV) promoter, and polyA. The circular full-length SARS-CoV-2 cDNA was constructed using the amplified fragments as CPER templates, did not require purification, and was directly transfected into HEK293T or HEK293-3P6C33 cells. Lastly, to obtain infectious virus particles with similar characteristics to wild-type viruses, the supernatant of the transfected cells was added to Vero E6 or Vero E6/TMPRSS2 cells and passaged continuously.

## 3. Construction Strategies of Biosafe Coronavirus Engineering Systems

### 3.1. Virus-like Particles (VLPs)

**Figure 4:**
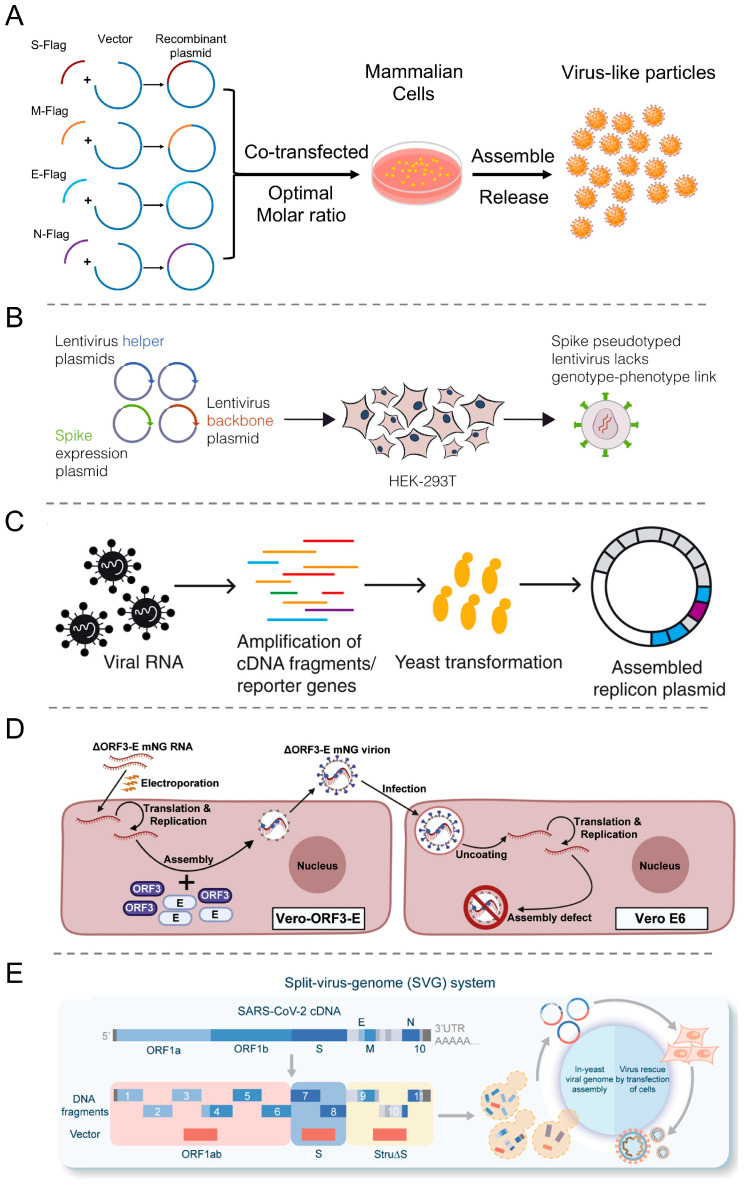
Construction strategies of biosafe coronavirus engineering systems. (**A**) Schematic outline of SARS-CoV-2 VLPs construction in mammalian expression system. Reproduced with permission from Ref. [80]. Copyright 2020, *Frontiers Media S.A.* (**B**) Traditional lentivirus pseudotyping method Reproduced with permission from Ref. [81]. Copyright 2023, *Cell Press.* (**C**) Optimized RNA production for SARS-CoV-2 replicons. Reproduced with permission from Ref. [82]. Copyright 2024, *AAAS.* (**D**) A trans-complementation system for SARS-CoV-2. Reproduced with permission from Ref. [83]. Copyright 2021, *Cell Press.* (**E**) Construction of SVG system based on SARS-CoV-2 genome. Reproduced with permission from Ref. [84]. Copyright 2022, *Science Press*.

The VLP are hollow particles formed by the self-assembly of one or more viral structural protein (Figure 4A). However, because of the lack of replication machinery, these VLPs cannot replicate or proliferate and thus have no risk of infection [51]. VLPs have a similar morphology to wild-type viruses and can specifically infect corresponding host cells [76]. Furthermore, these can display foreign antigen epitopes at a high density, which promotes the uptake of antigen-presenting cells, thereby efficiently inducing cellular immunity and neutralizing antibody reactions [77,78]. Therefore, VLPs are significant for studying high-risk viruses and have become safe and effective tools for vaccine development and virus-host interaction research [79].

Based on the presence or absence of lipid membranes, VLPs can be classified into enveloped and non-enveloped types. Non-enveloped VLPs (non-eVLPs) comprise single proteins, promote interactions between single or multiple viral structural proteins, and are relatively easy to produce and purify. However, the composition of enveloped VLPs (eVLPs) is relatively complex. The envelope of eVLPs originates from host cells and the outer surface comprises one or more glycoproteins [85,86,87]. These glycoproteins are important target antigens recognized by the immune system to produce neutralizing antibodies. In mammalian cells, the structural proteins of SARS-CoV-2 can be assembled into various types of eVLPs, such as S-E-M-N, S-E-M, E-M-N, and E-M [88].

Currently, VLPs are being developed by heterologous protein expression systems based on prokaryotic cells, yeast, insects, plants, and mammalian cells [89,90,91,92,93]. In these host expression systems, VLPs of SARS-CoV-2 and other CoVs can be successfully produced. Each expression system has its advantages and disadvantages [87,94]. For instance, the bacterial expression system is suitable for the rapid and efficient production of non-eVLPs but not for eVLPs [95]. This is because the system is prone to misfolding and cannot perform post-translational modifications, which is important for eukaryotic antigen expression. In contrast, the expression systems of yeast, insects, plants, and mammalian cells can all undergo post-translational modifications to generate both types of VLPs. It has been observed that the mammalian cell system is costly and time-consuming; however, the protein glycosylation of other systems is different from that of the mammalian cell system. For example, yeast undergoes high-mannose glycosylation, whereas insect glycosylation comprises α1,3-linked fucose residues and lacks terminal sialic acids [95,96].

### 3.2. Pseudovirus

Pseudoviruses are constructed by recombining the core/skeleton proteins and envelope proteins of different viruses [97] (Figure 4B). The pseudovirus genome is altered or modified to not express envelope proteins, typically by replacing the envelope protein gene with a reporter gene. Furthermore, to generate complete pseudoviruses, additional plasmids of envelope protein or stable cell lines are required. The commonly used skeleton viruses include human immunodeficiency virus (HIV), vesicular stomatitis virus (VSV), and murine leukemia virus (MLV) [98]. Pseudoviruses have the advantage of operability, low biological risk, and easy detection, making them suitable for research on highly infectious BSL-2 lab viruses [99]. Moreover, these have been employed to study the pathogenesis of highly infectious viruses such as SARS-CoV, MERS-CoV, and SARS-CoV-2, which is particularly important for understanding novel viruses [100,101,102].

The primary method of producing SARS-CoV-2 pseudoviruses is a lentivirus packaging system represented by HIV, which includes two/three-plasmid co-transfection systems. The two-plasmid system contains SARS-CoV-2 S plasmid and HIV skeleton plasmid lacking the envelope gene [103]. In the three-plasmid system, the HIV skeleton plasmid is divided into a packaging plasmid that expresses Gag and Pol proteins and transfer plasmid, containing the necessary reporter genes and *cis*-regulatory elements for HIV reverse transcription, integration, and packaging [104]. However, considering the safety issues of HIV, the simian immunodeficiency virus (SIV) system was developed and used by Moore et al. to construct pseudoviruses for screening anti-SARS-CoV compounds [105]. In addition, researchers have also used the feline immunodeficiency virus (FIV) packaging system to construct various pseudo-CoVs for studying virus receptor recognition, gene transduction, and treatment [106,107].

Furthermore, in comparison with the lentivirus packaging system, the VSV packaging system is widely used for pseudovirus construction due to its simple genome structure and broad host range. Moreover, there are only a few reports on the construction of pseudo-CoVs based on MLV packaging systems [108]. Marc et al. constructed pseudo-SARS-CoV-2 viruses via HIV, VSV, and MLV packaging systems through codon optimization and indicated very low yields of these infectious particles. In addition, other viral systems for chimeric expression of the SARS-CoV-2 S protein have also been investigated. For example, the NS1-deficient influenza A virus packaging system was used to express the SARS-CoV-2 S protein and served as a candidate vaccine in clinical trials [109]. Furthermore, for the expression of SARS-CoV-2 S protein, an adenovirus packaging system has also been used and has been indicated as a potential vaccine. Other packaging systems are also under development [110]. Yan et al. utilized bacteriophage systems, specifically MS2 bacteriophages, to generate SARS-CoV-2 pseudovirus by inserting N, E, and ORF1ab genes. The MS2 system effectively packaged 500 bp RNA; however, it had limitations in packaging longer RNA sequences [111].

As with other viruses, studying CoV requires some methods to detect the presence of the virus in infected cells and/or animal models. The recombinant CoVs (rCoVs) expressing reporter genes can solve this problem and achieve real-time tracking of viral infections by monitoring the reporter genes’ expression. Many studies have demonstrated the feasibility of using reverse genetic systems to generate rCoVs expressing reporter genes. Through genetic engineering, the structural or non-structural proteins of rCoVs are replaced by a reporter gene, such as IacZ gene, fluorescent protein gene, or luciferase reporter gene [112,113,114]. Hou et al. constructed two reporter viruses by replacing a 276-bp region in ORF7 with the green fluorescent protein (GFP) or a nanoluciferase (nLuc) gene fused with GFP. These reporter viruses were used to investigate the pathogenesis of SARS-CoV-2 and demonstrated limited cross-CoV neutralization in sera collected from SARS and COVID-19 patients [115].

### 3.3. Replicon

In virological research, another common strategy to improve biosafety is to delete viral structural genes and only retain replication-related genes to acquire a defective genome called replicon [116,117]. The replicon is a self-replicating sub-genome system that replaces the genes encoding viral structural proteins with reporter genes (Figure 4C). The NSPs required for virus replication and transcription can be encoded by replicons or expressed through other vectors. Generally, the CoV replicons–encoded RNA genomes have very similar structures, comprising 5′ and 3′ *cis*-acting elements required for virus replication, ORF 1ab encoding NSP, and the N gene necessary for effective RNA synthesis. Furthermore, replicons are the preferred tool for screening antiviral drugs against positive-stranded RNA viruses and studying the molecular mechanisms of virus replication [118]. The replicons of SARS-CoV, MERS-CoV, and SARS-CoV-2 have been successfully constructed [52,82,119].

Ge et al. constructed the SARS-CoV replicon by deleting the virus envelope protein-encoding genes S, E, and M to prevent virus production and secretion and retain the N gene due to its role in viral RNA synthesis [120]. Furthermore, approximately 60 nt were added upstream of the N gene for native expression. Moreover, in the replicon, the green fluorescent protein-lignin deaminase fusion (GFP-BlaR) gene was inserted between ORF 1ab and N. The expression of GFP-BlaR was driven by transcriptional regulatory sequences, and it was used for the identification and screening of replicon-containing cells [121]. SARS-CoV replicon was transfected into BHK-21 cells and subjected to hesperidin screening to obtain replicon-containing cells. Furthermore, Chen et al. constructed a MERS-CoV replicon carrying a reporter gene that primarily contained the neomycin phosphotransferase gene, renilla luciferase gene, and genes that encoded polyproteins pp1a/pp1ab and N protein. MERS-CoV replicon was transfected into BHK-21 cells and stable cell lines were screened [122].

The construction of SARS-CoV-2 replicons was also achieved by deleting structural protein genes, replacing the S gene with a reporter gene, and retaining the N gene and essential promoters. For in vitro transcription of T7 polymerase, its promoter at the 5′ end was used, whereas the polyA, hepatitis delta virus ribozyme, and terminator sequences at the 3′ end also facilitated transcription. Based on the SARS-CoV-2 Wuhan-Hu-1 strain genome, Liu et al. designed a replicon with both HIV LTR and T7 promoters at the 5′ end, which allowed co-expression of the viral replicon with HIV Tat protein or in vitro transcription through a T7 DNA-dependent RNA polymerase [123]. To ensure precise RNA production, Hammerhead and hepatitis delta virus ribozymes were incorporated at the 5′ and 3′ ends while retaining NSP1 and ORF10. Retaining N gene and transcriptional regulatory sequences allows effective replication and RNA expression. In addition, for monitoring translation, replication/transcription, and screening of stable cell lines, the firefly luciferase reporter gene and GFP-Blasticidin resistance fusion gene were inserted between NSP1 and NSP2, as well as between NSP16 and N, respectively [123].

### 3.4. Trans-Complementation System

Complementation is a naturally occurring genetic mechanism that can perform functional rescue on genomes with defects or mutations. In traditional complementation experiments, defective genomes or proteins are rescued by wild-type copies. In virological research, this system serves as a genomic tool for generating defective viruses for investigating protein function and virus particle assembly. Zhang et al. developed a non-virulent trans-complementation system that can simulate authentic virus replication under BSL-2 conditions [83] (Figure 4D). It has been observed that after E and ORF3 genes knockdown, the modified SARS-CoV-2 genome can only obtain single-round infectious particles (SRIPs) in the trans-complementation cell line expressing E and ORF3 proteins. The SRIPs were non-pathogenic in rodents and can be used for the screening of antiviral agents and high-throughput neutralizing antibodies. By using a trans-complementation system of VLPs and replicon, Su et al. generated SARS-CoV-2 SRIPs containing S protein variants [124]. The S gene of Wuhan-Hu-1 and Omicron BA.1 (BA.1) were co-expressed with the E and M genes, respectively, to obtain variant S-VLPs. Furthermore, WH1 strain replicon lacking S, E, M, and accessory genes have also been engineered to produce self-replicating RNA, resulting in SWH1- and SBA.1-based SRIPs. Compared with SWH1-based SRIPs, SBA.1-based SRIPs exhibited lower virus yield, replication, N protein expression, and infectivity.

### 3.5. Split-Virus-Genome (SVG) System

To advance SARS-CoV-2 research in the BSL-2 lab and ensure biosafety, Ma et al. designed and constructed an SVG system [84] (Figure 4E). In this system, the full-length SARS-CoV-2 cDNA was divided into three parts: S, ORF1ab, and Stru∆S. Then, the corresponding plasmids were co-transfected into 293T cells to rescue the reconstituted SARS-CoV-2 (rSARS-CoV-2). During rSARS-CoV-2 assembly, the packaging signal (PS) sequence was located at the 3′ end of the ORF1ab fragment, allowing the specific recognition of only this portion of the genome, as well as its packaging into virus particles. Therefore, rSARS-CoV-2 comprised all protein components and lacked all structural genes. This system can realistically simulate the various lifecycles of SARS-CoV-2 without producing progeny viruses and without proliferation risks. Moreover, it is a safe and universal research system, enabling researchers lacking high-level safety laboratory resources to conduct studies on highly infectious pathogenic microorganisms, thereby meeting the urgent needs of virological research.

## 4. Applications of Biosafety Coronavirus Engineering Systems

### 4.1. Mutant Strains

**Figure 5:**
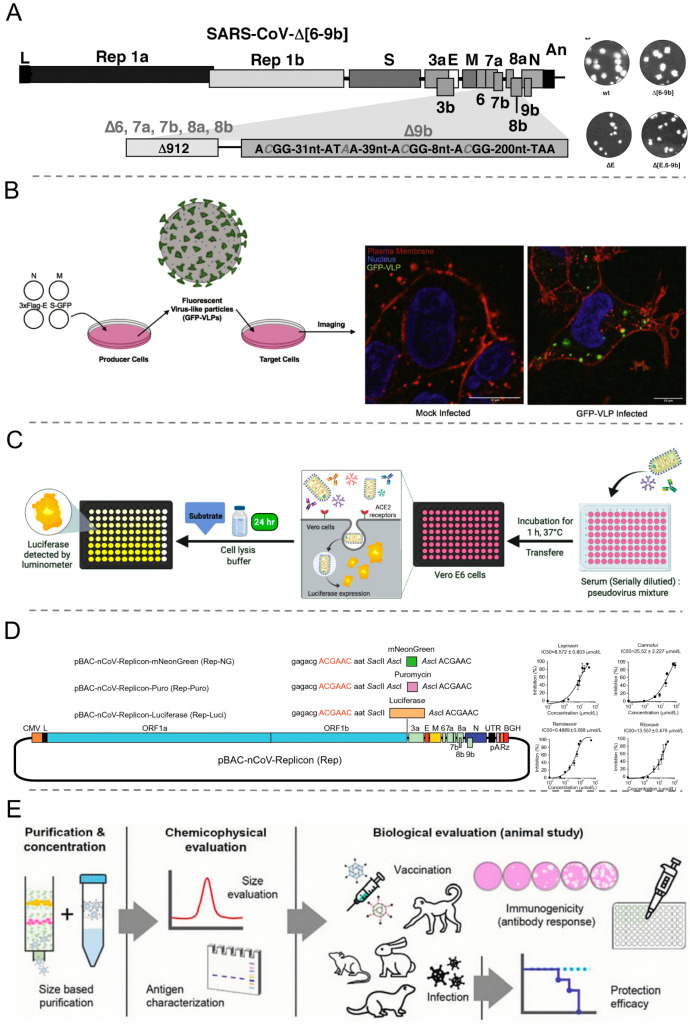
Applications of biosafe coronavirus engineering systems. (**A**) Evaluation of the pathogenicity of SARS-CoV deletion mutants by constructing cDNA. Reproduced with permission from Ref. [126]. Copyright 2008, *Elsevier.* (**B**) Monitoring the entry pathways with SARS-CoV-2 VLP. Reproduced with permission from Ref. [127]. Copyright 2021, *Elsevier.* (**C**) Graphical overview of protocol of pseudovirus-based neutralizing antibody evaluation. Reproduced with permission from Ref. [128]. Copyright 2020, *Frontiers Media S.A.* (**D**) SARS-CoV-2 replicon for drug screening system. Reproduced with permission from Ref. [54]. Copyright 2021, *Keai Publishing Itd.* (**E**) Physical and chemical evaluation of VLP-based vaccines before manufacture. Reproduced with permission from Ref. [129]. Copyright 2022, *Microbiological Society of Korea*.

Coronavirus pandemics have been accompanied by a high mutation rate. Therefore, the viruses’ infectivity and the effectiveness of existing prevention and treatment methods require re-evaluation. It is relatively difficult to isolate new mutant strains. Thus, generating pseudoviruses with specific mutation sites via targeted mutagenesis is of great significance for research. Using reverse genetics systems, site-specific mutations, deletions, or insertions can be introduced into the viral genome cDNA (Figure 5A). Furthermore, a comparison of the phenotypic differences between mutant strains and parental strains allows an in-depth analysis of the functions of targeted drugs and vaccines in virus replication, pathogenesis, and active genes [125].

The SARS-CoV-2 S protein has many mutations and is highly glycosylated. Li et al. constructed a pseudovirus comprising 80 S mutants and 26 glycosylation site mutations [130]. It was observed that RBD mutations and the degree of glycosylation directly affected the infectivity. Moreover, the mutant strains containing D614G were more infectious, while the absence of glycosylation in the N331 and N343 mutants greatly reduced infectivity. These findings are essential for vaccine development and antibody screening. The SARS-CoV-2 N protein is relatively conserved and highly phosphorylated, which is necessary for genome RNA packaging and virus release [131]. Recently, it was revealed that the deletion of the 500–532 locus in the SARS-CoV-2 NSP1 decreased the viral load and serum IFN-β level [132]. In addition, NSP6 mutation inhibits lipid droplet channels, thereby reducing the BA.1 virulence [133]. The study of single or multiple accessory protein–deleted strains showed that the accessory protein played an important role in the virulence and incidence rate of SARS-CoV-2.

### 4.2. Virus Life Cycle

VLPs and pseudoviruses are valuable tools for studying the entry pathways and cell line preferences of CoVs (Figure 5B). In their study, Kumar et al. fused HibiT protein tags with the genes of different structural proteins to monitor VLP generation and entry [134]. Ma et al. used dual-fluorescence technology to track the endocytic pathways of SARS-CoV-2 in various respiratory epithelial cells [135]. Furthermore, Simmons et al. established pseudoviruses to evaluate the impact of SARS-CoV S protein cleavage by different proteases on entry into host cells via the plasma membrane or endosomal pathways [136]. For example, cathepsin L cleaved the T678 site of the SARS-CoV S protein, allowing it to enter host cells through the endosomal pathway. Matsuyama et al. further confirmed that, in wild-type viruses, the efficiency of entry via the plasma membrane pathway was >100 times greater than via the endosomal pathways [137]. In the study of virus replication, replicons with specific gene deletions play an important role. The SARS-CoV-2 replicon constructed by Jin et al. had key mutations in NSP12 and NSP14, which reduced RNA replication/transcription ability [54]. Almazán et al. created three defective SARS-CoV replicons, among which the absence of NSP14, NSP15, and NSP16 led to defects in three RNA processing enzymes, resulting in minimal expression of the replicon [125]. In addition, Wang et al. revealed that the replicon lacking only NSP16 still exhibited replication ability. Therefore, it was hypothesized that the observed difference may be due to the presence of accessory proteins [138].

### 4.3. Neutralizing Antibody Evaluation and Antiviral Drugs Screening

Due to the limited number of BSL-3 laboratories, the evaluation of neutralizing antibodies largely relies on pseudoviruses. The interaction of pseudo-SARS-CoV-2 expressing chimeric S protein with the ACE2 receptor facilitates its entry into host cells. The cell lines used for neutralization assays are usually natural or ACE2-expressing transfected cells, such as Huh7, Vero, 293T, BHK21, and HeLa (Figure 5C). Overexpression of TMPRSS2 protease can enhance the susceptibility of ACE2-expressing cells to SARS-CoV and SARS-CoV-2. Zettl et al. utilized the SARS-CoV-2 pseudovirus to assess the neutralizing antibody titer of COVID-19 patients in convalescence, and most patients showed low titers [41]. Furthermore, Cao et al. employed SARS-CoV-2 pseudovirus and identified 14 effective neutralizing antibodies from 60 convalescent patients [139]. Wang et al. found that patients infected with wild-type SARS-CoV-2 exhibited low neutralizing activity against the B.1.1.7 variant pseudovirus [140]. For screening antiviral drugs, the replicon with reporter genes is an ideal model. For instance, Jin et al. used the replicon with the luciferase reporter gene to verify the inhibitory effect of remdesivir [54] (Figure 5D). Moreover, Ge et al. used replicons with GFP and developed a high-throughput system to screen 7035 small molecule compounds and identified 7 effective drugs against SARS-CoV-2, significantly accelerating the progress of drug screening [121]. In addition, Ju et al. employed the trans-complementary system for high-throughput screening of 377 natural products and identified salinomycin, tubeimoside I, monensin sodium, lycorine chloride, and sodium nigericin as candidate drugs for targeting virus replication or entry [141].

### 4.4. Vaccine Development

Vaccine development is crucial for combating highly pathogenic infectious diseases. The pseudoviruses and VLPs-based vaccines can simulate the structure and antigen characteristics of viruses and have been highly regarded for their safety, tolerance, and efficiency (Figure 5E). In their experiment, Qin et al. inserted the RBD gene of SARS-CoV (a common target) into the adeno-associated virus (AAV) genome to prepare a vaccine [142]. Gao et al. induced monkeys to produce antibodies by expressing the S1, M, and N genes of SARS-CoV using adenovirus vectors [143]. Furthermore, Fougeroux et al. employed the SpyTag/SpyCatcher system to connect the *Acinetobacter* phage vector with the SARS-CoV-2 RBD domain to produce a nano-vaccine [144]. This vaccine can induce antibodies in mice and is currently under clinical trial. Hu et al. developed liposome nano-VLPs combined with the S1 subunit of SARS-CoV-2, which successfully triggered cellular and mucosal immunity in mice [145]. Moreover, based on polylactic acid-glycolic acid, Lin et al. engineered hollow spherical nanoparticles by incorporating the RBD region of MERS-CoV and encapsulating them with a stimulator of interferon gene (STING) to enhance immune response [146].

## 5. Future Outlook

Following the outbreaks of SARS-CoV and MERS-CoV, the emergence of SARS-CoV-2 was the third major outbreak caused by the CoVs. The high pathogenicity of these CoVs requires careful treatment in laboratory research. However, limitations in the number and distribution of BSL-3 facilities severely constrain their scientific exploration. Therefore, the development of engineering systems that can improve biosafety is crucial. VLPs and pseudoviruses contain viral structural proteins, which can be rapidly prepared and play important roles in viral entry and tropism, the neutralization of antibody preparation, and vaccine development. Furthermore, replicons are also a valuable tool for the screening of antiviral drugs, as well as the evaluation of replication-related genes and molecular mechanisms. Additionally, trans-complementary and SVG systems contained all viral components and defective genomes, demonstrating enormous potential in extensive virological research. However, even with the development of multiple engineering systems for studying CoVs, there are still many limitations due to the large size of the CoV genome and its susceptibility to mutations, which hinder pathogenesis evaluation and vaccine development. With the rapid development of gene editing technology, bioinformatics, and artificial intelligence, the precise modification of genes, analysis of virus structures, and research on transmission patterns have become easier and more efficient. Researchers are expected to reduce the infectivity and pathogenicity of virulent viruses through more precise and rapid intervention methods to promote the safe research of CoVs and better respond to unknown new infectious diseases.

## Data Availability

Not applicable.
